# Randomized controlled trials of iron chelators for the treatment of cardiac siderosis in thalassaemia major

**DOI:** 10.3389/fphar.2014.00217

**Published:** 2014-09-23

**Authors:** A. John Baksi, Dudley J. Pennell

**Affiliations:** NIHR Cardiovascular Biomedical Research Unit, Royal Brompton & Harefield NHS Foundation Trust & Imperial College LondonLondon, UK

**Keywords:** cardiac siderosis, iron, chelation trials, T2* imaging, thalassaemia

## Abstract

In conditions requiring repeated blood transfusion or where iron metabolism is abnormal, heart failure may result from accumulation of iron in the heart (cardiac siderosis). Death due to heart failure from cardiac iron overload has accounted for considerable early mortality in β-thalassemia major. The ability to detect iron loading in the heart by cardiovascular magnetic resonance using T2* sequences has created an opportunity to intervene in the natural history of such conditions. However, effective and well tolerated therapy is required to remove iron from the heart. There are currently three approved commercially available iron chelators: deferoxamine, deferiprone and deferasirox. We review the high quality randomized controlled trials in this area for iron chelation therapy in the management of cardiac siderosis.

## INTRODUCTION

In conditions of iron overload, either due to recurrent blood transfusion or abnormal iron metabolism, iron accumulation within organs can have fatal consequences. Beta-thalassemia major (TM) is a condition of abnormal hemoglobin resulting in profound anemia that requires lifelong regular blood transfusion, typically from the age of 2 years. As a consequence of the repeated transfusions and the body’s inability to clear excess iron, iron can build up within tissues. The accumulation of iron within the heart (cardiac siderosis) can result in heart failure and this has been the commonest cause of death in TM. The development of iron chelators offered a solution to this problem, but long-term experience showed persistently high and premature cardiac mortality. This has been related in large part to conventional measures of iron loading such as serum ferritin and liver iron concentration being poor surrogates of iron loading in the heart. It is possible to have high levels of iron loading in the liver without significant cardiac loading and vice versa. Consequently cardiac siderosis may not be identified until late, when it presents with heart failure that is associated with a very poor prognosis.

Over recent decades, the observation that iron reduces magnetic resonance (MR) signal has lead to the development of targeted MR sequences to assess the tissue iron concentrations in the liver and the heart. The use of cardiovascular magnetic resonance (CMR) to quantify the parameter T2^∗^ as a measure of myocardial iron concentration has been particularly important as it remains the only technique available for this purpose (Anderson et al., 2001; Baksi and Pennell, 2014). T2^∗^ assessment has now been robustly validated,(Kirk et al., 2009; Carpenter et al., 2011) is well established and is widely used (Carpenter et al., 2013). In addition to being able to identify iron loading in the heart and liver, the T2^∗^ technique can be used to assess the efficacy of iron chelators and to guide therapy. Prior to the development of CMR T2^∗^, establishing the efficacy of iron chelation therapies on myocardial siderosis was not possible with any accuracy in the absence of cardiac biopsy. Little data was available to accurately guide the application of iron chelators or assess their safety and patient compliance with treatment.

There are few randomized control trials (RCT) of iron chelation for cardiac siderosis. In addition to the paucity of data, the robustness of the presented data is variable. We describe and review the published trials, relating them to the recent guidelines for the management of β-thalassemia major by the American Heart Association (AHA; Pennell et al., 2013). The efficacy of the iron chelators for liver iron is not discussed in this review, and we focus on heart failure due to cardiac siderosis as the main driver of mortality. We only discuss RCTs using T2^∗^ MR as an endpoint, because it remains the only validated technique to measure myocardial iron. Myocardial T2^∗^ is considered “normal” when >20 ms. Cardiac iron loading is considered “present” with T2^∗^ <20 ms, and is considered severe with T2^∗^ <10 ms because the rate of heart failure increases dramatically below this threshold (Anderson et al., 2001).

## COMMERCIALLY AVAILABLE IRON CHELATORS

Currently, there are three commercially available products with FDA and EU approval for iron chelation in the clinical setting. As well as varying in the mode of administration, there are significant differences between the three chelators with regard to efficacy, tolerability, and patient compliance.

### DEFEROXAMINE

The first iron chelator approved for clinical use was deferoxamine. The greatest drawback with this agent is that due to poor gut uptake, it needs to be given parenterally (either subcutaneously, or in heart failure due to iron overload by intravenous infusion). The short half life results in typical administration for 8–12 h per day for at least 5 days of the week. This has a profound impact on patient compliance and considerable work has been done to provide devices for subcutaneous drug delivery. Once severe cardiac iron loading is present, months to years of careful therapy is needed to clear the iron. Even when used as a continuous intravenous infusion, deferoxamine only clears myocardial iron at 5% per month (Pennell et al., 2006; Tanner et al., 2007). Side effects of therapy include deafness, visual disturbance and growth retardation as well as a high frequency of skin reactions.

### DEFERIPRONE

Deferiprone was licensed around 30 years later than deferoxamine. Deferiprone is rapidly absorbed from the gastrointestinal tract and is therefore taken orally, usually three times a day. The oral formulation has significant compliance advantages over deferoxamine. Significant side effects include agranulocytosis, gastrointestinal disturbance and arthropathy. Given the risk of neutropenia, weekly monitoring of the neutrophil count is advised. Deferiprone is commonly used in combination with deferoxamine for enhanced iron clearance.

### DEFERASIROX

The most recent of the chelators is deferasirox. This is taken orally with rapid absorption and can be taken once daily. Adverse events are typically gastro-intestinal upset, rash and raised creatinine. More serious adverse events include Fanconi syndrome, and eye and ear toxicity. Monitoring of renal and hepatic function are recommended for deferasirox.

## RANDOMIZED CONTROLLED TRIAL DATA COMPARING IRON CHELATORS IN THE HEART

There have been three RCTs examining the role of the iron chelators in cardiac siderosis using T2^∗^ CMR as an endpoint. The first RCT compared oral deferiprone monotherapy against subcutaneous deferoxamine (Pennell et al., 2013). The achieved dose of deferiprone was 92 mg/kg per day and deferoxamine was 43 mg/kg for 5.7 days per week. The improvement in myocardial T2^∗^ was significantly greater for deferiprone than deferoxamine (27% vs. 13%; *P* = 0.023). Left ventricular (LV) ejection fraction (EF) increased significantly more in the deferiprone treated group (3.1% vs. 0.3% absolute units; *P* = 0.003). This RCT established that deferiprone was superior to deferoxamine over 1 year for the removal of cardiac iron, and the improvement of LV EF in patients with asymptomatic myocardial siderosis. A further report from this trial also showed that right ventricular EF also improved more in the deferiprone patients, than with deferoxamine (Alpendurada et al., 2010).

The second RCT compared combination treatment of deferiprone plus deferoxamine against deferoxamine alone in 65 patients with myocardial T2^∗^ from 8 to 20 ms representing a range from severe to mild cardiac siderosis (Pennell et al., 2006). There were significant improvements in the combined treatment group compared with deferoxamine alone in myocardial T2^∗^ (ratio of change in geometric means +50% versus +24%; *P* = 0.02). The combined group also showed significantly great improvement in absolute LV EF (2.6% versus 0.6%; *P* = 0.05), and absolute endothelial function (8.8% versus 3.3%; *P* = 0.02). A further report from this trial showed that right ventricular EF also improved more in the combined group, than with deferoxamine alone (Alpendurada et al., 2012).

The third RCT (CORDELIA trial) compared oral deferasirox against subcutaneous deferoxamine (Pennell et al., 2014). This was a prospective, randomized comparison of deferasirox (target dose 40 mg/kg per day) vs. subcutaneous deferoxamine (50–60 mg/kg per day for 5–7 days/week) for myocardial iron removal in 197 thalassemia major patients with myocardial siderosis (T2^∗^ 6–20 ms) and no signs of cardiac dysfunction. The geometric mean of the myocardial T2^∗^ improved with deferasirox from 11.2 ms to 12.6 ms at 1 year (+12%) and with deferoxamine from 11.6 ms to 12.3 ms (+7%). The between-arm ratio of the geometric means was 1.056 with the 95% confidence intervals of 0.998 and 1.133). The lower boundary was greater than the pre-specified margin of 0.9, establishing non-inferiority of deferasirox vs. deferoxamine (*P* = 0.057 for superiority of deferasirox). Mean LVEF remained stable and within the normal range after 1 year of treatment with deferasirox (66.9–66.3%) and deferoxamine (66.4–66.4%). The change in mean LVEF after 1 year was not different between the two treatments (*P* = 0.54).

There are two meta-analyses of trials reporting cardiac iron, but they both have significant limitations in including trials using non-T2^∗^ assessment technology and also non-randomized trials (Mamtani and Kulkarni, 2008; Xia et al., 2013). This demonstrates the paucity of data in the literature and emphasises the need to focus on the three gold standard randomized controlled trials described above.

## OTHER DATA AND GUIDELINES FOR USE

### DEFERIPRONE VERSUS DEFEROXAMINE

The superiority of deferiprone monotherapy over standard deferoxamine therapy has been suggested by retrospective and cross-sectional studies (Anderson et al., 2002; Pepe et al., 2006, 2011). RCT data indicate that deferiprone is the most efficacious of these agents for reducing myocardial iron, clearing cardiac iron at nearly double the rate of deferoxamine, and also improving EF, an effect not observed with deferoxamine (Pennell et al., 2013). This remains the only prospective randomized control trial of deferiprone monotherapy compared to deferoxamine, and further data would be welcome, as recommended by a Cochrane review (Fisher et al., 2013). The AHA guidelines on heart management in thalassaemia major, indicate that deferiprone monotherapy is useful in patients with cardiac siderosis, and it is also suitable for patients with reduced LVEF or asymptomatic LV dysfunction (Carpenter et al., 2013).

### COMBINED DEFERIPRONE AND DEFEROXAMINE VERSUS DEFEROXAMINE MONOTHERAPY

The RCT by Tanner showed that combined treatment with deferiprone and deferoxamine was superior to deferoxamine alone in removing cardiac iron and improving EF. These findings accord with other trial data (Daar and Pathare, 2006; Kattamis et al., 2006; Kolnagou and Kontoghiorghes, 2006; Christoforidis et al., 2007) and a Cochrane review which again suggested that further trial data would be helpful (Fisher et al., 2013). The AHA Guidelines indicate that combined treatment is commonly used where cardiac iron loading is moderate to severe or when LV function is impaired (Carpenter et al., 2013).

### MONOTHERAPY WITH DEFERASIROX

The efficacy of deferasirox monotherapy with regard to its ability to reduce myocardial iron has now been established in a prospective randomized control trial of good size (Alpendurada et al., 2012). Non-inferiority to deferoxamine was demonstrated. Also of note was LV function remained stable during treatment with both deferasirox and deferoxamine, and consequently, the AHA guidelines does not recommend the use of deferasirox as first line treatment where there is established depression of LV EF, or in those with severe iron loading (Carpenter et al., 2013). Data investigating the impact of combination therapy with deferasirox and deferiprone would be direct comparison of interest, as would a trial of deferasirox against deferiprone.

### ACUTE HEART FAILURE DUE TO IRON OVERLOAD

Continuous intravenous deferoxamine is the default consensus treatment for acute heart failure due to myocardial iron toxicity. At present, there is very limited RCT data in this setting. A small trial of iron chelation in heart failure found both deferoxamine monotherapy and combination therapy with deferiprone and deferoxamine to be effective in improving LVEF and myocardial T2^∗^ (Porter et al., 2013). A non-randomized trial found combined treatment to be effective in severe cardiac siderosis with reduced LV EF (Tanner et al., 2008).

## CONCLUSION

The assessment of myocardial iron by T2^∗^ CMR is now established as fundamental to the best practice management of thalassaemia. This has enabled the investigation of which chelation treatment regime is suitable for myocardial iron loading. Unfortunately, many published studies have suboptimal design and are essentially observational with low patient numbers, and do not contribute significantly to the evidence base for determining cardiac treatment. T2^∗^ CMR is pivotal to inform management and prolong survival. This is reflected no more strongly than in that data showed a 71% reduction in mortality in the UK β-thalassemia major population consequent on the introduction of routine screening for cardiac siderosis by CMR T2^∗^(Modell et al., 2008).

Whilst all the available iron chelation therapies appear to be effective if given in high enough doses with patient compliance, from the available evidence, deferiprone appears to have superior efficacy compared to deferoxamine, and the effect of deferoxamine is superior when combined with deferiprone compared to deferoxamine alone. Additionally, deferasirox appears to have equivalent efficacy to deferoxamine. Each of these therapies has advantages and disadvantages and tailoring therapy to individual patients is important to optimize outcome. Despite the current evidence base, further larger, well-designed, randomized controlled trials are desirable, and greater data on compliance is needed. Well targeted application of these therapies in combination with T2^∗^ CMR will improve the prognosis of thalassaemia major patients.

## AUTHOR CONTRIBUTIONS

A. John Baksi and Dudley J. Pennell contributed to data review and manuscript preparation.

## Conflict of Interest Statement

A. John Baksi has no commercial or financial relationships that could be construed as a potential conflict of interest. Dudley J. Pennell is a shareholder in Cardiovascular Imaging Solutions (London, UK) and consultant to Siemens. He is also a consultant to ApoPharma, Novartis, Shire and Bayer.
